# Expression and characterization of a GH43 endo-arabinanase from *Thermotoga thermarum*

**DOI:** 10.1186/1472-6750-14-35

**Published:** 2014-04-30

**Authors:** Hao Shi, Huaihai Ding, Yingjuan Huang, Liangliang Wang, Yu Zhang, Xun Li, Fei Wang

**Affiliations:** 1 College of Chemical Engineering, Nanjing Forestry University, Nanjing 210037, China; 2 Jiangsu Key Lab of Biomass-Based Green Fuels and Chemicals, Nanjing 210037, China

**Keywords:** Arabinan, Arabinose, Endo-arabinanase, *Thermotoga thermarum*

## Abstract

**Background:**

Arabinan is an important plant polysaccharide degraded mainly by two hydrolytic enzymes, endo-arabinanase and α-L-arabinofuranosidase. In this study, the characterization and application in arabinan degradation of an endo-arabinanase from *Thermotoga thermarum* were investigated.

**Results:**

The recombinant endo-arabinanase was expressed in *Escherichia coli* BL21 (DE3) and purified by heat treatment followed by purification on a nickel affinity column chromatography. The purified endo-arabinanase exhibited optimal activity at pH 6.5 and 75°C and its residual activity retained more than 80% of its initial activity after being incubated at 80°C for 2 h. The results showed that the endo-arabinanase was very effective for arabinan degradation at higher temperature. When linear arabinan was used as the substrate, the apparent *K*_*m*_ and *V*_*max*_ values were determined to be 12.3 ± 0.15 mg ml^−1^ and 1,052.1 ± 12.7 μmol ml^−1^ min^−1^, respectively (at pH 6.5, 75°C), and the calculated *k*_*cat*_ value was 349.3 ± 4.2 s^−1^.

**Conclusions:**

This work provides a useful endo-arabinanase with high thermostability andcatalytic efficiency, and these characteristics exhibit a great potential for enzymatic conversion of arabinan.

## Background

Lignocellulosic biomass, the most abundant renewable carbon resources in the biosphere, has exhibited valuable industrial applications in many fields, such as pulp and paper, food processing, detergent, textile, synthetic biology, organic synthesis, pharmaceuticals and bioenergy production [1,2]. Arabinan, a polysaccharide constituent of hemicellulose, is composed of α-1,2- and/or α-1,3-linked to a α-1,5-linked L-arabinofuranosyl backbone residues [3-5]. Arabinan mainly consists of L-arabinose residues, existing in rhamnogalacturonan regions of pectins in the cell walls of several plants [6]. Microbial enzymes including hydrolases have represented an important part in industry due to their various favorable properties [7]. To generate L-arabinose from arabinan, two major hydrolytic enzymes, endo-arabinanase and α-L-arabinofuranosidase, are essential to the synergetic action. Endo-arabinanase (EC 3.2.1.99) can hydrolyze the arabinan α-1,5-linked L-arabinofuranosyl backbone to produce arabino-oligosaccharides or L-arabinoses, whereas α-L-arabinofuranosidase (EC 3.2.1.55) is capable of thoroughly hydrolyzing arabino-oligosaccharides and arabinan side chain substitutions to produce L-arabinoses [3]. In addition, exo-arabinanase (EC 3.2.1.-) is able to release terminal arabinose (non-reducing end with net retention of the anomeric configuration) or arabino-oligosaccharides from arabinan [8,9]. Based on the amino acid sequence similarities, endo-arabinanases and exo-arabinanases have been classified into glycoside hydrolase (GH) family 43 and GH93 family, respectively, whereas α-L-arabinofuranosidases belong to five GH families (GH3, 43, 51, 54, 62) (http://www.cazy.org/). These arabinan-degrading enzymes from different GH families have presented various applications in industrial processes such as food technology, nutritional medical research and organic synthesis [4].

A few reports about the endo-arabinanases from bacteria and fungi have been published so far [5,6,8,10-19]. Among all the studies above, only a novel hyperthermophilic endo-arabinanase from *Thermotoga petrophila* was biophysically characterized and the three-dimensional (3D) structure was released [1,17]. Although the genome of hyperthermophilic *Thermotoga thermarum* DSM 5069 has been sequenced, no information is currently available on the endo-arabinanase. In this study, a putative GH43 β-xylosidase from *Thermotoga thermarum*, Tth Abn, was cloned and expressed. Its biochemical characterization and application in arabinan degradation were also investigated. Upon the evaluation of substrate specificity, it was interesting to find that the recombinant enzyme was an endo-arabinanase, which has not yet been reported.

## Results

### Amino acid sequence of Tth Abn

**Figure 1:**
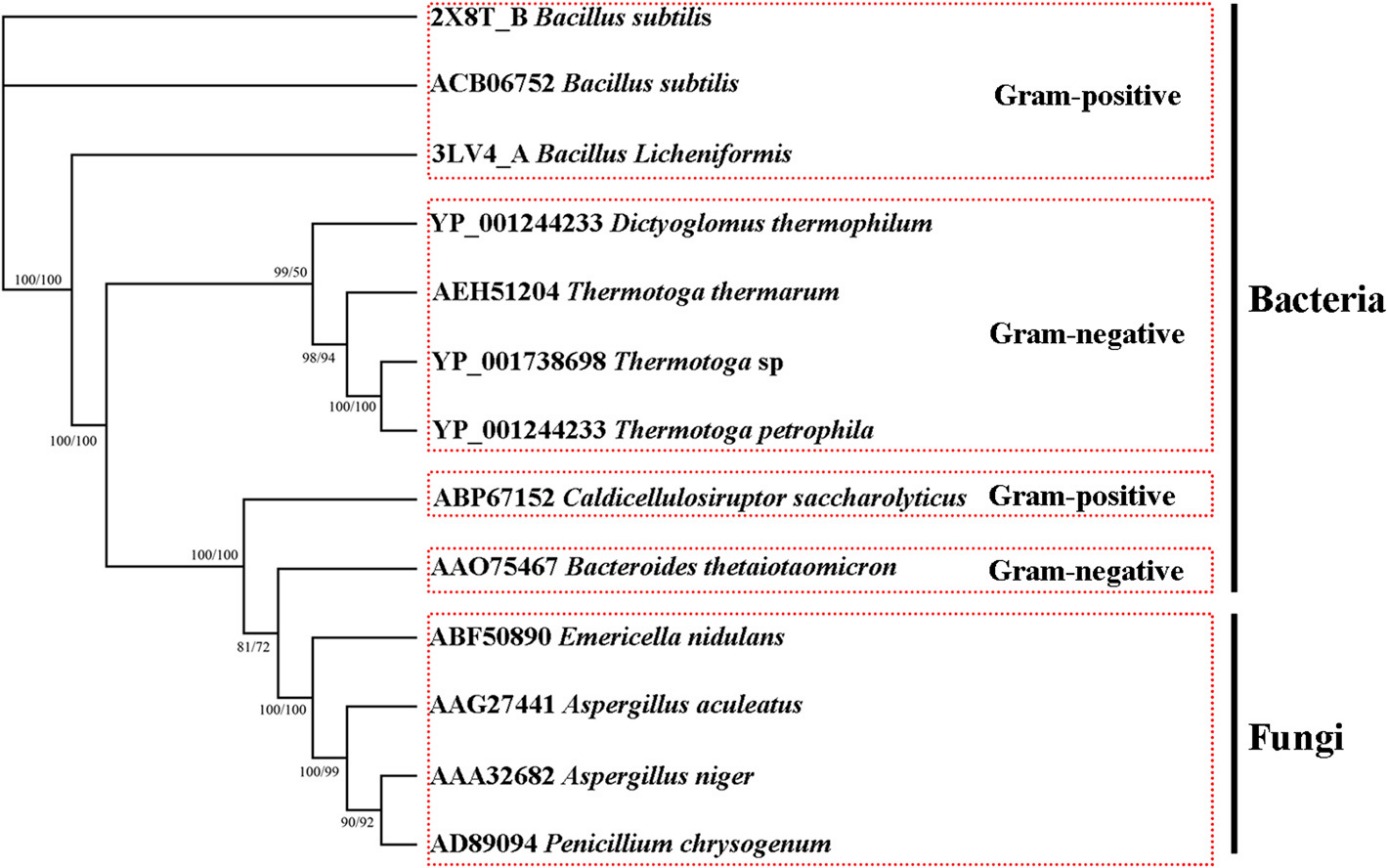
**Neighbor-Joining (NJ) and Maximum-Parsimony (MP) trees resulted from analysis of endo-arabinanase with 13 amino acid sequences.** Numbers on nodes correspond to percentage bootstrap values for 1000 replicates. The former confidence number in the Figure 1 is for NJ tree and the latter is for MP tree.

The *Tth abn* gene isolated from the *T. thermarum* genome is 1,437 bp in length coding for 479 amino acids. There is a signal peptide sequence (32 amino acids, 96 bp) in Tth Abn when analyzed by SignalP 4.0 (http://www.cbs.dtu.dk/services/SignalP/). Therefore, only the DNA fragment of 1,341 bp was amplified. Amino acid sequence of Tth Abn in GenBank is described as a putative β-xylosidase. However, it contains putative domains of α-L-arabinofuranosidase (EC 3.2.1.55), endo-arabinanase (EC 3.2.1.99) and β-xylosidase (EC 3.2.1.37) through the BLAST. In this study, the provisional β-xylosidase was finally confirmed as an endo-arabinanase through the biochemical analysis. Tth Abn exhibited 64% identity to the β-xylosidase from *Thermotoga* sp*.* RQ2 (GenBank accession number YP_001738698) and *Thermotoga petrophila* RKU-1 (GenBank accession number YP_001244233), 59% identity to putative β-xylosidase *Dictyoglomus thermophilum* H-6-12 (GenBank accession number YP_002251442), and 50% identity to the endo-arabinanase from *B. subtilis* Abn2 H318A mutant (GenBank accession number YP_002251442 2X8T). To gain insights into the evolutionary relationships among endo-arabinanases, the phylogenetic trees with 13 candidate sequences were constructed using the NJ and MP methods, respectively, which both supported the same topological structures as shown in Figure 1. Phylogenetic analysis indicated that Tth Abn from *T. thermarum* had a close relationship with an endo-arabinanase from *T. petrophila* and a putative β-xylosidase from the same genus *T*. sp.

**Figure 2:**
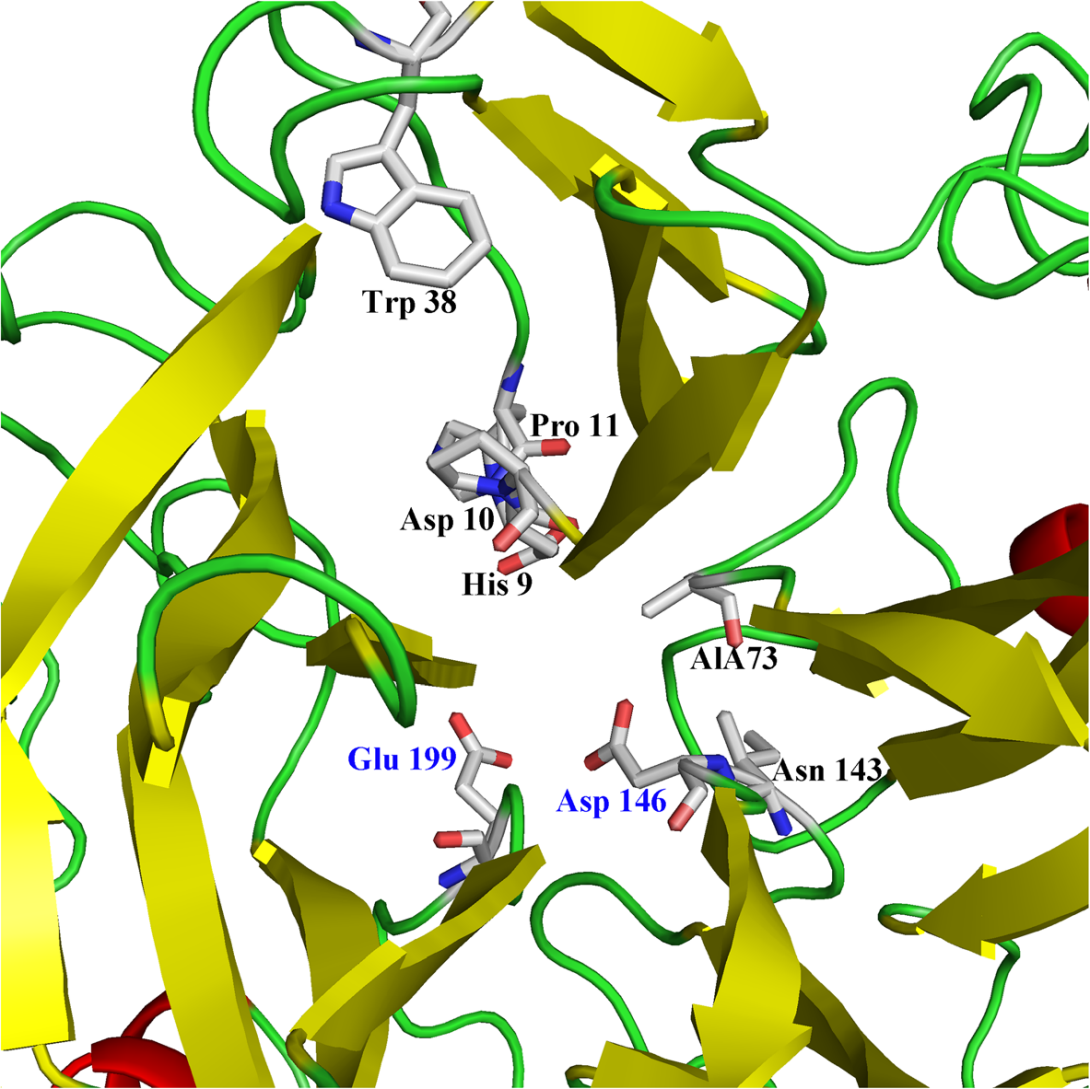
**Predicted three-dimensional structure of Tth Abn.** The potential catalytic amino acid residues are Asp^146^ and Glu^199^.

Homology modeling revealed that Tth Abn had a similar 5-fold β-propeller structure as the *B. subtilis* endo-arabinanase Abn2 H318A mutant [11] (Figure 2). Both sequence comparison and homology modeling indicated that Asp^146^ and Glu^199^ residues were the catalytic nucleophile and proton donor, respectively [11]. Furthermore, there are other conserved amino acid residues adjacent to the catalytic center, His^9^, Asp^10^, Pro^11^, Trp^38^, Ala^73^ and Asn^143^, which may play some roles in catalytic activity.

### Expression and purification of Tth Abn

**Figure 3:**
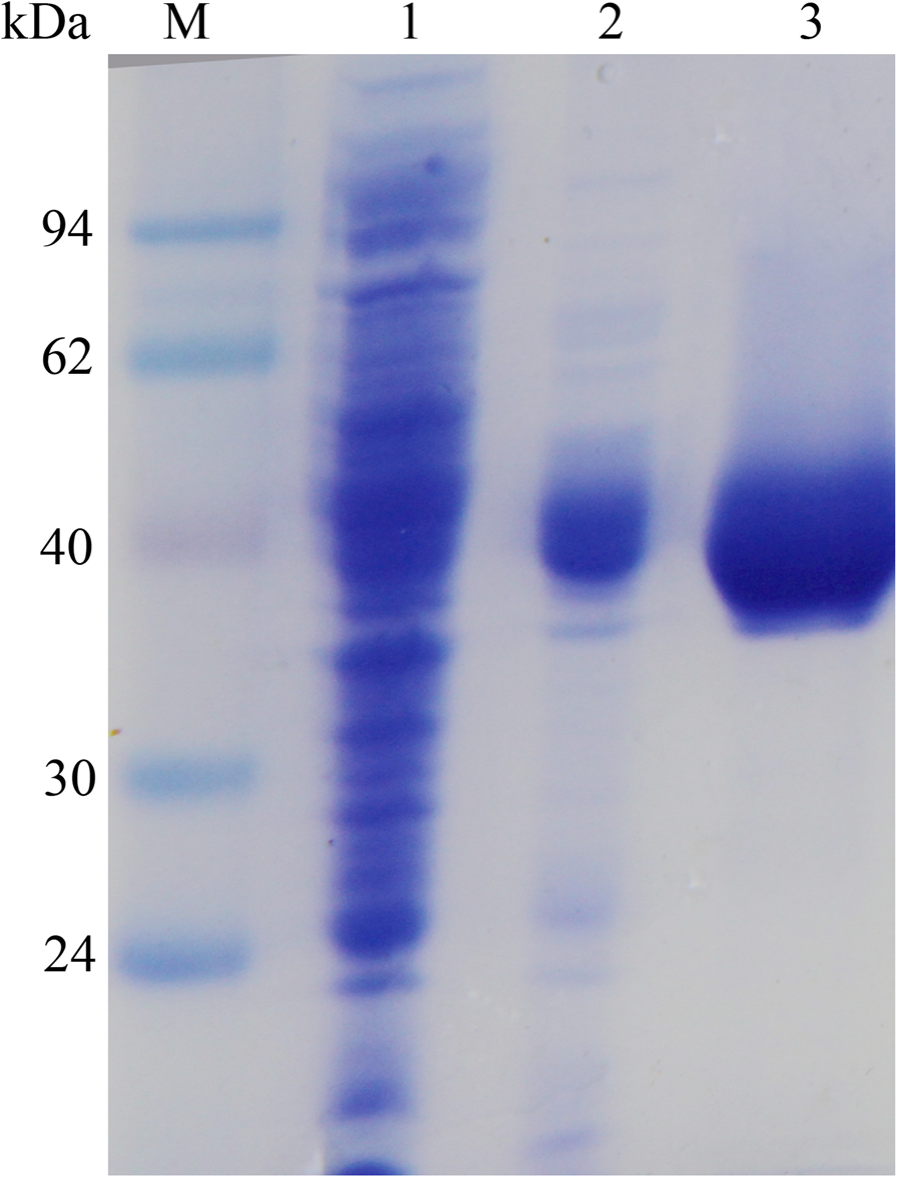
**SDS-PAGE analysis of recombinant Tth Abn in *E. coli *BL21 (DE3).** Lane M: protein marker, lane 1: cell-free extract of *E. coli* BL21 (DE3) harboring recombinant plasmids, lane 2: the purified Tth Abn eluted with 0.2 M imidazole buffer, lane 3: the purified Tth Abn eluted with 0.4 M imidazole buffer.

Mature protein coding 447 amino acids was successfully expressed in the cytoplasmatic fraction of *E. coli* BL21 (DE3). Then, the protein in the cell-free extract was purified by more than 95% homogeneity after heat treatment and affinity chromatography (Additional file 1: Table S1). Finally, the purified fusion enzyme showed a single band on a SDS-PAGE gel with an estimated molecular weight of 36 kDa (Figure 3). To examine the oligomerization state of the enzyme, size exclusion chromatography was performed. The native protein formed monomer in solution with a calculated MW 35,622 Da according to the calibration curve of the gel filtration column.

### Biochemical properties of Tth Abn

Enzymatic characteristics of purified recombinant Tth Abn were determined and summarized in Tables 1, 2 and 3. Substrate specificity was assayed with different substrates (Table 1). It was interesting to find that Tth Abn was active towards linear arabinan, debranched arabinan and sugar beet arabinan, but there was no activity towards 1,4-β-D-mannan, galactan, *p*-Nitrophenyl-β-D-xylopyranoside (*p*NPX), and *p*-nitrophenyl-α-L-arabinofuranoside (*p*NPAF). These results indicated that the enzyme only exhibited arabinanase activity.

**Table 1:** Specific activity of Tth Abn on various substrates

**Substrate**	**Specific activity (μmol arabinose min**^**−1**^ **mg**^**−1**^**)**
Linear arabinan	237.7 ± 10.0
Debranched arabinan	199.7 ± 6.8
Sugar beet arabinan	42.5 ± 1.5
1,4-β-D-Mannan	ND
Galactan	ND
*p*-Nitrophenyl-α-L-arabinofuranoside	ND
*p*-Nitrophenyl-β-D-xylopyranoside	ND

ND: not detected. Values shown were the mean of triplicate experiments, and the variation about the mean was below 5%.

**Table 2:** Effects of cations and chemical reagents on the activity of purified Tth Abn

**Cations**^**a**^**/Chemical reagents**^**b**^	**Residual activity (%)**
Control	100 ± 0.6
Mg^2+^	99 ± 1.2
Zn^2+^	14 ± 0.3
Mn^2+^	128 ± 2.9
Ba^2+^	113 ± 3.6
Ca^2+^	113 ± 1.7
Al^3+^	0
Cu^2+^	0
Co^2+^	82 ± 1.7
Ni^2+^	31 ± 0.5
Tween 60	133 ± 2.5
Tris	41 ± 0.4
SDS	111 ± 1.8

^a^Final concentration, 1 mM. ^b^Final concentration, 0.05% Tween 60 and Tris, 0.1% SDS. Values shown were the mean of triplicate experiments, and the variation about the mean was below 5%.

**Table 3:** **Characteristics of endo-arabinanases from *T. thermarum *** **DSM 5069 and other microorganisms**

**Strain**	***K***_***m ***_**(mg ml**^**−1**^**)**			***V***_***max ***_**(μmol ml**^**−1**^ **min**^**−1**^**)**			***k***_***cat ***_**(s**^**−1**^**)**			***k***_***cat***_***/K***_***m ***_**(ml mg**^**−1**^ **s**^**−1**^**)**			**Optimal temp (°C)**	**Reference**
*Thermotoga thermarum*	^a^12.3 ± 0.15	^b^3.3 ± 0.12	^c^28.6 ± 0.88	^a^1,052.1 ± 12.7	^b^510.9 ± 17.5	^c^164.0 ± 4.6	^a^349.3 ± 4.2	^b^169.6 ± 5.9	^c^54.4 ± 1.6	^a^28.4 ± 0.4	^b^51.4 ± 0.1	^c^1.9 ± 0.0	75	This study
*Caldicellulosiruptor saccharolyticus*	^b^18.1			ND			^b^49.8			^b^2.8			75	[3]
*Bacillus licheniformis*	^b^19 ± 0.2			ND			^b^161 ± 1.2			^b^8.47 ± 0.16			35	[5]
*Bacillus subtilis*	^a^2.0 ± 0.24			^a,d^250 ± 1.2			ND			ND			50	[20]
*Thermotoga petrophila*	^b^19.9 ± 4.7			^b^478.2 ± 63.6			ND			ND			73	[1]
*Aspergillus aculeatus*	^b^66			11			ND			ND			50	[16]

ND: not determined. Data were carried out with linear arabinan (a), debranched arabinan (b), or sugar beet arabinan (c) as substrate. Values shown were the mean of triplicate experiments, and the variation about the mean was below 5%. d: μmol mg^−1^ min^−1^.

**Figure 4:**
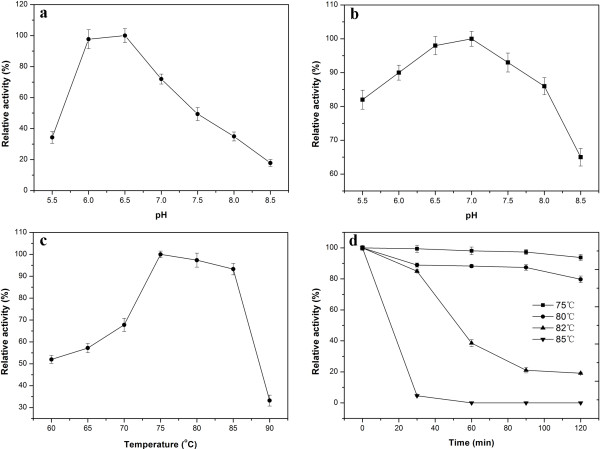
**Effects of pH and temperature on the activity and stability of the recombinant Tth Abn endo-arabinanase. a**. Optimal pH of the Tth Abn. **b**. pH stability of the Tth Abn. **c**. Effect of temperature on Tth Abn activity. **d**. The thermostability of the Tth Abn. The residual activity was monitored, and the maximum activity was defined as 100% **(a, c)** or initial activity was defined as 100% **(b, d)**. Values shown were the mean of triplicate experiments, and the variation about the mean was below 5%.

Effect of pH on Tth Abn activity was determined in 50 mM imidazole-potassium buffer ranging from pH 5.5 to 8.5 (Figure 4a). Tth Abn was found to exhibit an optimum activity at pH 6.5 and was able to retain more than 95% of its initial activity at 70°C for 1 h (Figure 4b). As a function of temperature, the enzyme displayed the highest activity at 75°C and retained more than 90% of its maximum activity even at 85°C after 10 min incubation (Figure 4c). Incubations at different temperatures to determine the enzyme’s thermal stability were also carried out. As shown in the result, the enzyme kept nearly its initial activity at 75°C after 2 h incubation, and could still retain more than 80% of its original activity at 80°C for 2 h (Figure 4d).

Effects of metal ions and chemicals on Tth Abn activity were shown in Table 2. In various assays, the enzyme activity was apparently stimulated by 1 mM Mn^2+^, Ca^2+^, Ba^2+^, or chemical reagents 0.05% Tween 60 and 0.1% SDS. On the contrast, Al^3+^, Cu^2+^ and Zn^2+^ could inhibit the enzyme activity evidently. Kinetic studies in the presence of three arabinans as the substrates at optimum temperature and pH allowed the determination of the Michaelis-Menten parameters as shown in Table 3.

### Degradation of arabinan by Tth Abn

**Figure 5:**
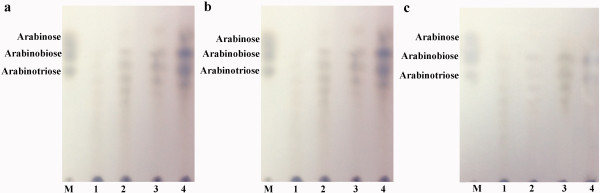
**Analysis of linear arabinan and debranched arabinan hydrolyzed by Tth Abn endo-arabinanase.** The products of the reaction were examined with TLC. M: arabinose, arabinobiose and arabinotriose. **a**. Lane 1, 2, 3, 4: linear arabinan samples (2%, wt/vol) incubated with 1 μg Tth Abn endo-arabinanase for 0.5 h, 1 h, 2 h and 3 h. respectively. **b**. Lane 1, 2, 3, 4: debranched arabinan samples (2%, wt/vol) incubated with 1 μg Tth Abn endo-arabinanase for 0.5 h, 1 h, 2 h and 3 h, respectively. **c**. Lane 1, 2, 3, 4: sugar beet arabinan samples (2%, wt/vol) incubated with 1 μg Tth Abn endo-arabinanase for 0.5 h, 1 h, 2 h and 3 h, respectively.

Catalytic ability of Tth Abn on arabinan was investigated by analyzing the digestion products of linear , debranched and sugar beet arabinans (Figure 5a,b,c). Clearly from the result, the end products for hydrolysis of linear and debranched arabinans were arabinose, arabinobiose and arabinotriose after degradation for 3 h. However, it displayed very weak catalytic ability towards the hydrolysis of sugar beet arabinan.

## Discussion

Enzymatic hydrolysis of arabinose-linked hemicellulose in plant cell wall requires endo-arabinanase, arabinofuranosidase and arabinoxylan arabinofuranohydrolase, which are capable of releasing arabinosyl oligomers and L-arabinose from arabinose-containing polysaccharides [20].

We reported an endo-arabinanase (Tth Abn) from the hyperthermophilic bacterium *T. thermarum* here which was originally predicted as a β-xylosidase yet possessed the capability of arabinan depolymerization. Similar results have been published that endo-arabinanase from *Thermotoga petrophila* RKU-1 and *B. subtilis* were confirmed as endo-arabinanases through biophysical characterizations although once were predicted as β-xylosidases [1,20]. It is well known that hydrolases from GH43 family utilize a single displacement mechanism, which results in an inverted anomeric configuration for hydrolysis products [8]. Amino acid sequence alignment and homology modeling indicated that Asp^146^ and Glu^199^ in Tth Abn were the catalytic nucleophile (base) and proton donor, respectively. The result is also supported by the fact that aspartic acid and glutamic acid residues are the catalytic nucleophile and proton donor in all members of GH43 family (http://www.cazy.org). Residue Asp^10^ may act as a pKa modulator and maintain the correct alignment of the general acid residue relative to the substrate [21]. Residues Asp^10^, Asp^146^ and Glu^199^ located in the deep cavity at the centre of the β-propeller are also found in all members of GH32, 43, 62 and 68 [22]. As the consequence of the flexibility reduction of the polypeptide chain, residue Pro^11^ may contribute to the high thermal stability of the enzyme [23].

Phylogenetic analysis and enzymatic properties showed that Tth Abn was distant with the endo-arabinanases from *Bacillus subtilis*, *Bacillus licheniformis* and *Aspergillus aculeatus*[4,5,19]. However, it revealed a close relationship with *T. thermarum* and *T. petrophila*, indicating that they share the similar properties [1,17]. Other endo-arabinanases from genus *Thermotoga* including *T. thermarum* endo-arabinanase have not yet been studied. Since there is 64% amino acid sequences similarity between endo-arabinanases *T. thermarum* and *T. petrophila* and is also inferred by the experimental data shown in Table 3, it is confirmed that Tth Abn could be an endo-arabinanase with some specific properties.

Tth Abn exhibited good thermostability when incubated at 75° for 2 h. Endo-arabinanase from Gram-negative bacterium *T. petrophila* RKU-1 has been reported to have a pH optimum at 6.5 and retained 95% of initial activity at 90°C for 10 h [1]. While Gram-positive bacteria *B. subtilis* and *B. licheniformis* endo-arabinanases show optimum activity at near-neutral pH at 60°C and 35°C, respectively [4]. Endo-arabinanase *Aspergillus aculeatus* from fungus displays optimum activity at pH 5.5 and 50°C [16]. In general, endo-arabinanases from bacteria exhibit the near-neutral pH optima, while those from fungi display the acid pH optima. The enzyme activities regarding pH from bacteria and fungi are also consistent with their inherent properties.

Cations and chemical reagents exhibited different effects on the activity of the endo-arabinanase Tth Abn. With the evidence that one Ca^2+^ ion was observed in the catalytic cavity of *Bacillus subtilis* about 5 Å below the catalytic carboxylates and the presence of ions in an equivalent location in other arabinanases [22].

Compared to other endo-arabinanases, Tth Abn demonstrated the highest *V*_*max*_ value among all the known endo-arabinanases from thermophile and hyperthermophile when linear arabinan was used as the substrate. The catalytic efficiency (*k*_*cat*_/*K*_*m*_) of endo-arabinanase Tth Abn on debranched arabinanwas approximately 6-fold and 18-fold higher than *B. licheniformis* and *Caldicellulosiruptor saccharolyticus*, respectively (Table 3). In addition, catalytic efficiency for linear, debranched and sugar beet arabinans existed obvious differences (linear > debranched > sugar beet). This may be caused by substrate’s composition and structure. As we know, a typical sugar composition of linear arabinan is: arabinose: galactose: rhamnose: galacturonic acid = 97.5: 0.4: 0.1: 2.0. For debranched and sugar beet arabinans, the contents of arabinose are lower than linear arabinan, and sugar beet arabinan has more substitutions attached to backbone while 1,2- and 1,3-α-L-arabinofuranosyl branch units of debranched arabinan have been all removed.

The fact that Tth Abn could hydrolyze linear and debranched arabinan into oligomers arabinose, arabinobiose and arabinotriose implied that the Tth Abn had endo-arabinanase activity but no exo activity. Only a few endo-arabinanases especially that from hyperthermophile have been reported to possess the function in generating arabino-oligosaccharides [1,17]. Although it was insensitive to the branched arabinan (eg. sugar beet arabinan), Tth Abn could be used together with other arabinan-degrading enzymes in synergistic reaction for the hydrolysis of branched ones.

## Conclusions

In this study, a useful endo-arabinanase (Tth Abn) from *T. thermarum* DSM 5069 was expressed in *E. coli* with desirable features, such as high thermostability, catalytic efficiency, and hydrolytic reaction at high temperature. This is easily envisioned that Tth Abn endo-arabinanase exhibits a great potential for enzymatic conversion of arabinan through synergetic action with other arabinan-degrading enzymes.

## Methods

### Substrates

Linear arabinan, debranched arabinan, sugar beet arabinan, 1,4-β-D-mannan and galactan were purchased from Megazyme (Wicklow, Ireland). *p*NPX and *p*NPAF were purchased from Sigma Chemical Co., Ltd. (St. louis, USA).

### Bacterial strains and growth conditions

*E. coli* Top10 (Novagen, San Diego, USA) was used for routine molecular cloning work and *E. coli* BL21 (DE3) (Novagen) was employed as the host for the expression of the recombinant Tth Abn. *E. coli* strains, both were grown in Luria-Bertani (LB) medium. Ampicillin (100 μg ml^−1^, Shanghai, China) and isopropyl-β-D-thiogalactopyranoside (IPTG, 0.5 mM, Dalian, China) were added when necessary.

### Construction of plasmids and strains

DNA manipulations were carried out according to standard methods [24]. Restriction enzymes and high-fidelity Ex-Taq DNA polymerase were purchased from Takara Biotechnology Co. Ltd. (Dalian, China) and used according to the manufacturer’s instructions. DNA was extracted from agarose gels with BIOMIGA Gel Extraction Kit (Shanghai, China). Sequencing was performed using ABI 3730 DNA analyzer (Applied Biosystems, Foster City, USA). PCR amplifications were conducted using high-fidelity Ex-Taq DNA polymerase, and the resulting products were purified with BIOMIGA PCR Purification Kit (Shanghai, China).

The coding sequence of *Tth abn* gene was amplified from *T. thermarum* genomic DNA using primers 5’-GGAATTCCATATGGTATTCAACTGGGCAACTGTACAC-3’ (forward) and 5’- CCGCTCGAGATCTTCGATCCGAACTCCCCAG-3’ (reverse). The primers contained restrictions sites *Nde*I and *Xho*I (underlined) for forward and reverse primers, respectively. The amplified DNA fragment was digested with *Nde*I and *Xho*I, and inserted into the corresponding sites in plasmid pET-20b (Novagen) to produce pET-20b-*Tth abn*. The correctness of the insert was confirmed by DNA sequencing.

### Expression and purification of recombinant Tth Abn

*E. coli* BL21 (DE3) cells harboring recombinant plasmids were grown (200 rpm, 37°C) in LB medium (200 ml) with appropriate antibiotic selection. When the OD_600_ reached 0.6 ~ 0.8, the expression of Tth Abn was induced by the addition of 0.5 mM IPTG and the culture was further incubated (150 rpm, 25°C, 12 h) . Cells were harvested by centrifugation (10000 rpm, 4°C, 5 min). The pellet was washed twice with 20 mM Tris–HCl buffer (pH 8.0), and re-suspended in 5 ml of 5 mM imidazole, 0.5 mM NaCl, and 20 mM Tris–HCl buffer (pH 7.9). All steps were carried out at 4°C. The cell extracts after sonication were heat treated at 70°C for 30 min, and subsequently cooled in an ice bath. After centrifugation (15000 g, 4°C, 20 min), the resulting supernatant was loaded onto a 1 ml nickle affinity column (Novagen) and the bounded proteins were eluted by discontinuous imidazole gradient (30–1000 mM). The fractions that contained Tth Abn were dialyzed overnight against storage buffer (20 mM Na-phosphate, 50 mM NaCl, 10% glycerol, pH 7.0) and then were kept at −80°C until further use. The analysis of production and purity of the enzymes were determined by a 12% SDS-PAGE gel [25] using broad range molecular weight markers (MBI Fermemtas, Burlington, Canada) as standards. The protein concentration was determined by Bradford method using bovine serum albumin (BSA) as a standard [26]. Oligomerization state of Tth Abn was analyzed by size exclusion chromatography on a AKTA*FPLC*™ (GE Healthcare Life Sciences, New Jersey, USA) system with a Superdex 200 10/30 GL column as described by Zhang et al. [27].

### Biochemical characterization

Unless otherwise stated, all the enzyme-catalyzed reactions were performed at 75°C for 10 min in 50 mM imidazole-potassium buffer (pH 6.5) containing 0.1 μg enzyme and 0.4% (w/v) linear arabinan. The total reaction system was 0.2 ml. The reducing sugar content after hydrolysis of the polysaccharides was determined by the 3,5-dinitrosalicylic acid method [28] with L-arabinose as the standard. One enzyme activity unit was defined as the amount of enzyme producing 1 μmol of arabinose per minute. All assays were performed in triplicate.

Optimum temperature and pH for enzymatic activity of the Tth Abn were examined with linear arabinan 0.4% (w/v) as substrate for 10 min. The effect of pH on the activity was tested at pH 5.5 to 8.5 in 50 mM imidazole-potassium buffer at 75°C. The effect of temperature was assayed at temperatures ranging from 60°C to 95°C in 50 mM imidazole-potassium buffer at pH 6.5. The pH stability was determined from pH 5.5 to 8.5 at 75°C for 1 h. Thermostability of the enzyme was estimated by incubating the diluted solution of enzyme at different temperatures (75°C, 80°C, 82°C and 85°C). Samples were removed after different time intervals (30 min, 60 min, 90 min, and 120 min) and kept in ice for 10 min.

Enzymatic activity was also examined in the presence of metal ions and chemical reagents. Magnesium chloride (MgCl_2_), zinc sulfate (ZnSO_4_), barium chloride (BaCl_2_), nickel sulfate (NiSO_4_), manganese chloride (MnCl_2_), calcium chloride (CaCl_2_), cobalt chloride (CoCl_2_), copper sulfate (CuSO_4_) or aluminum sulfate (Al_2_(SO_4_)_3_) were used at a concentration of 1 mM, and the chemical reagents Tween 60, Tris or sodium dodecyl sulfate (SDS) were added at 0.05%, 0.05% and 0.1%, respectively in the reaction mixture. The enzyme was incubated with each cation or reagent at 80°C for 2 h before the addition of linear arabinan 0.4% (w/v) to initiate the enzyme reaction. The activity of the enzyme without adding chemical reagent or metal ion was defined as 100%. The kinetic parameters, apparent *K*_*m*_ and *V*_*max*_ values were calculated according to the Lineweaver-Burk method at optimum pH and temperature using linear arabinan as substrate at concentrations ranging from 1.25 to 30 mg ml^−1^.

### Substrate specificity and degradation

The abilities of the enzyme to hydrolyze the natural and modified substrates, including linear arabinan, debranched arabinan, sugar beet arabinan, 1,4-β-D-mannan, galactan, *p*NPX and *p*NPAF, were measured at 75°C for various time (0.5 h, 1 h, 2 h and 3 h). The reaction mixture (600 μL) contained 2% substrate (wt/vol) and 1 μg enzyme in 50 mM imidazole-potassium buffer (pH 6.5). The reaction was stopped by cooling the mixture at 4°C in a water bath. Thin-layer chromatography (TLC) was applied to monitor the degradation of linear arabinan using arabinose, arabinobiose and arabinotriose as the standard markers. TLC was performed using G plates (Qingdao, China) with a mobile phase containing *n*-butanol/acetic acid/water (3:1:1). The sugars that degraded were detected using the orcinol/concentrated sulfuric acid reagent [29].

### Bioinformatics analysis

Multiple sequence alignment was conducted using Clustal X2 [30]. Phylogenetic relationships were deduced using the Neighbor-Joining (NJ) and Maximum-Parsimony (MP) methods as performed in PAUP 4.0 [31]. Homology modeling was performed using Swiss Model [32-34]. The crystal structure of a GH43 endo-arabinanase from *Bacillus subtilis* (PDB entry code: 2X8T) was used as three-dimensional template for restraint-based modeling as implemented in SPDBV_4.04_PC.

### Nucleotide sequence accession number

GenBank accession number of the *T. thermarum* DSM5069 endoarabinanase is AEH51204.

## Competing interests

The authors declare that they have no competing interests.

## Authors’ contributions

HS carried out the cloning and expression and drafted the manuscript. HD, YH, LW, and XL helped to purify and characterize the Tth Abn. YZ helped to revise the manuscript. FW directed the overall study and revised the manuscript. All authors have read and approved the final manuscript.

## Supplementary Material

Additional file 1 Supplementary Information. Click here for file
