# Supplementary material for: Expression and characterization of a GH43 endo-arabinanase from Thermotoga thermarum
Source: BMC Biotechnol. 2014 Apr 30;14:35. doi: 10.1186/1472-6750-14-35 (PMC4021227; doi:10.1186/1472-6750-14-35)
Supplement: Additional file 1 — Supplementary Information. [file 1472-6750-14-35-S1.doc]

Supplementary Information

**BMC Biotechnology**

**Expression and characterization of a GH43 endo-****arabinanase from *Thermotoga thermarum***

Hao Shi^1,2^, Huaihai Ding^1,2^, Yingjuan Huang^1,2^, Liangliang Wang^1,2^, Yu Zhang^1,2^, Xun Li^1,2^, Fei Wang^1,2^*

^1^College of Chemical Engineering, Nanjing Forestry University, ^2^Jiangsu Key Lab of Biomass-Based Green Fuels and Chemicals, Nanjing 210037, China

*Corresponding Author

E-mail: hgwf@njfu.edu.cn, Tel: +86-25-85427649; Fax: +86-25-85427649

**Additional file 1: Table S1** Purification of the recombinant Tth Abn endo-arabinanase.

| Purification step | Total volume (mL) | Total activity  (μ mol min^-1^) | Total protein  (mg) | Specific activity (μ mol mg^-1^ min^-1^) | Recovery  (%) | Purification (fold) |
| --- | --- | --- | --- | --- | --- | --- |
| Crude extract^a^ | 10 | 2548 | 98 | 26 | 100 | 1 |
| Heat treatment^b^ | 10 | 2128 | 19 | 112 | 83.5 | 4.3 |
| Ni affinity chromatography^c^ | 1 | 1904 | 8 | 238 | 74.7 | 9.2 |

^a^ The recombinant strain was grown in LB medium (200 ml) with 100 μg ampicillin/ml at 37^o^C to OD_600_ 0.8 and was incubated further with isopropyl-β-thiogalactopyranoside (IPTG) for 12 h at 25^o^C. The cells were harvested by centrifugation at 10,000 g for 15 min at 4^o^C and resuspended in 10 ml imidazole buffer (10 mL of 5 mM imidazole, 0.5 mM NaCl, and 20 mM Tris-HCl buffer, pH 7.9), followed by sonication.

^b^ The cell extracts after sonication were heat treated at 70^o^C for 30 min, and then cooled in an ice bath, centrifuged at 15,000 g for 20 min at 4^o^C and the supernatant was kept.

^c^The obtained supernatants were loaded on to an immobilized metal affinity column (Novagen, USA), and eluted with 0.4 M imidazole, 0.5 M NaCl, and 20 mM Tris-HCl buffer (pH 7.9).
